# Analysis of the clinical profile and treatment efficiency of hyperlipidemic acute pancreatitis

**DOI:** 10.1186/s12944-024-02057-5

**Published:** 2024-03-08

**Authors:** Weidong Zhou, Qinfu Liu, Zhaojun Wang, Liying Yao, Jian Chen, Xiaojun Yang

**Affiliations:** 1 The First Clinical Medical College of Ningxia Medical University, Yinchuan, Ningxia 750004 China; 2 General Hospital of Ningxia Medical University, Yinchuan, Ningxia 750004 China

**Keywords:** Risk factors, Triglyceride, Severity, Plasma exchange, Prognosis

## Abstract

**Background:**

The incidence of hyperlipidemic acute pancreatitis (HLAP) has been increasing annually. However, population-based morbidity assessments need to be updated. Early, rapid, and effective lipid-lowering may minimize pancreatic injury and improve clinical prognosis. It is essential to choose the proper treatment. However, treatment options for HLAP are controversial, and there is no uniform treatment protocol.

**Methods:**

In this retrospective study, 127 patients with hyperlipidemic severe acute pancreatitis (HL-SAP) were registered from January 2018 to December 2022 at the General Hospital of Ningxia Medical University. Medical and radiological records of hospitalized patients were collected to determine clinical features, severity, complications, mortality, recurrence rate, and treatment. Risk factors for HL-SAP were analyzed using multifactorial logistic regression. A propensity score matching method was used to compare the clinical outcomes of standard and plasma exchange therapies.

**Results:**

In this research, the prevalence of HLAP increased about 1.6 times, and the prevalence of HL-SAP was 50.60%. HL-SAP occurs most often in people between the ages of 30 and 39. Amylase exceeded 110 U/L in 84.3% of patients and 330 U/L in only 47.2%. 83.5% of HL-SAP patients had fatty livers and high body mass index (BMI). A total of 48.0% of patients experienced organ failure, ICU treatment (55.1%), recurrence (33.1%), and death (21.3%). Between the hyperlipidemic group and the biliary group in terms of age, gender, BMI, fatty liver, pleural effusion, abdominal constriction syndrome (ACS), multiple organ dysfunction syndrome (MODS), length of hospital, medical costs, morbidity and mortality, triglyceride, cholesterol, creatinine, blood glucose, D-dimer, amylase, albumin, lactate dehydrogenase, serum phosphorus, serum calcium, oxygenation index, and recurrence rate were statistically significant (*P* < 0.05). High BMI (*P* = 0.0038, odds ratio (OR) = 1.336, 95%CI: 0.99–1.804), high C-reactive protein (CRP) (*P* = 0.022, OR = 1.011, 95%CI: 1.003–1.019), low calcium (*P* = 0.003, OR = 0.016, 95%CI. 0.001–0.239), low albumin (*P* = 0.012, OR = 0.045, 95%CI: -0.062-0.192), and high D-dimer (*P* = 0.041, OR = 0.619, 95%CI: 0.053–2.510) were risk factors for HL-SAP, according to multifactorial logistic regression analysis. Adjusted for propensity score matching (PSM), Serum triglyceride (TG) was significantly lower in both the standard treatment (*P* < 0.001) and plasma exchange (*P* < 0.001) groups at 48 h compared with the initial test after the attack. Clearance (83.20% ± 0.0% vs. 84.4% ± 0.0%, *P* = 0.531), length of hospital stay (19.9 ± 4.9 vs. 19.8 ± 11.1, *P* = 0.092), and death (26.3% vs. 23.6%, *P* = 0.791) showed no difference between the two groups. However, the difference in medical costs(*P* = 0.039)between the two groups was statistically significant.

**Conclusion:**

The incidence of HLAP exhibited a significant increase, remarkable severity, recurrent trend, and mortality. High BMI, high CRP, low calcium, low albumin, and high D-dimer are risk factors for HL-SAP. Compared with standardized treatment, plasma exchange does not improve the prognosis of HL-SAP patients, and standardized treatment is equally effective, safe, and low-cost in early treatment.

## Introduction

Acute pancreatitis is characterized by inflammation in the pancreas and is regarded as a common digestive illness that may result in hospitalization. Diagnosing acute pancreatitis relies upon observing characteristic abdominal imaging abnormalities [1–3]. The etiology of acute pancreatitis differs by region worldwide. Currently, the most frequently observed causes in most studies are gallstones (40–70%) and alcohol consumption (25–35%) [4]. Recent studies have indicated a growing prevalence of hyperlipidemic acute pancreatitis (HLAP) in Asia [5]. According to multiple studies, HLAP accounts for 2–5% of global acute pancreatitis cases, but this percentage has reached 10–15% in Asian populations [6]. A study conducted in Beijing showed that 10.36% of patients with HLAP developed acute hemorrhagic necrotizing pancreatitis [7]. Furthermore, there is evidence indicating that HLAP may be linked to a more intense clinical progression and increased mortality compared to other forms of pancreatitis. Patients in remote areas with severe acute pancreatitis who exhibit pancreatic necrosis or organ failure have mortality rates as high as 40% [4, 8–10].

Regarding the pathogenesis of HLAP, the widely accepted Havel theory suggests that triglycerides (TG) and their free fatty acids (FFA) are closely related to the development of HLAP, and some studies have also confirmed the toxic effects of TG and FFA on the pancreas [11]. Therefore, early, rapid, and effective elimination of high concentrations of TG and FFA from the body may help to reduce pancreatic damage and improve clinical prognosis. Effective treatments for lowering serum TG levels include lipid-lowering drugs, insulin, heparin, low-molecular heparin, and blood purification techniques, including haemoperfusion, plasma exchange, and continuous renal replacement therapy [12, 13]. However, standardized guidelines for the treatment of HLAP have not been developed. Currently, there is still controversy about whether to adopt conventional or plasma exchange therapy for patients with HLAP after admission.

Early diagnosis of pancreatitis and identification of risk factors for severe disease are essential for targeted treatment of HLAP. This study aimed to develop safe, effective, and low-cost therapies for patients with hyperlipidemic severe acute pancreatitis (HL-SAP) by summarising the clinical profile of HL-SAP.

## Materials and methods

### Study population

**Fig. 1:**
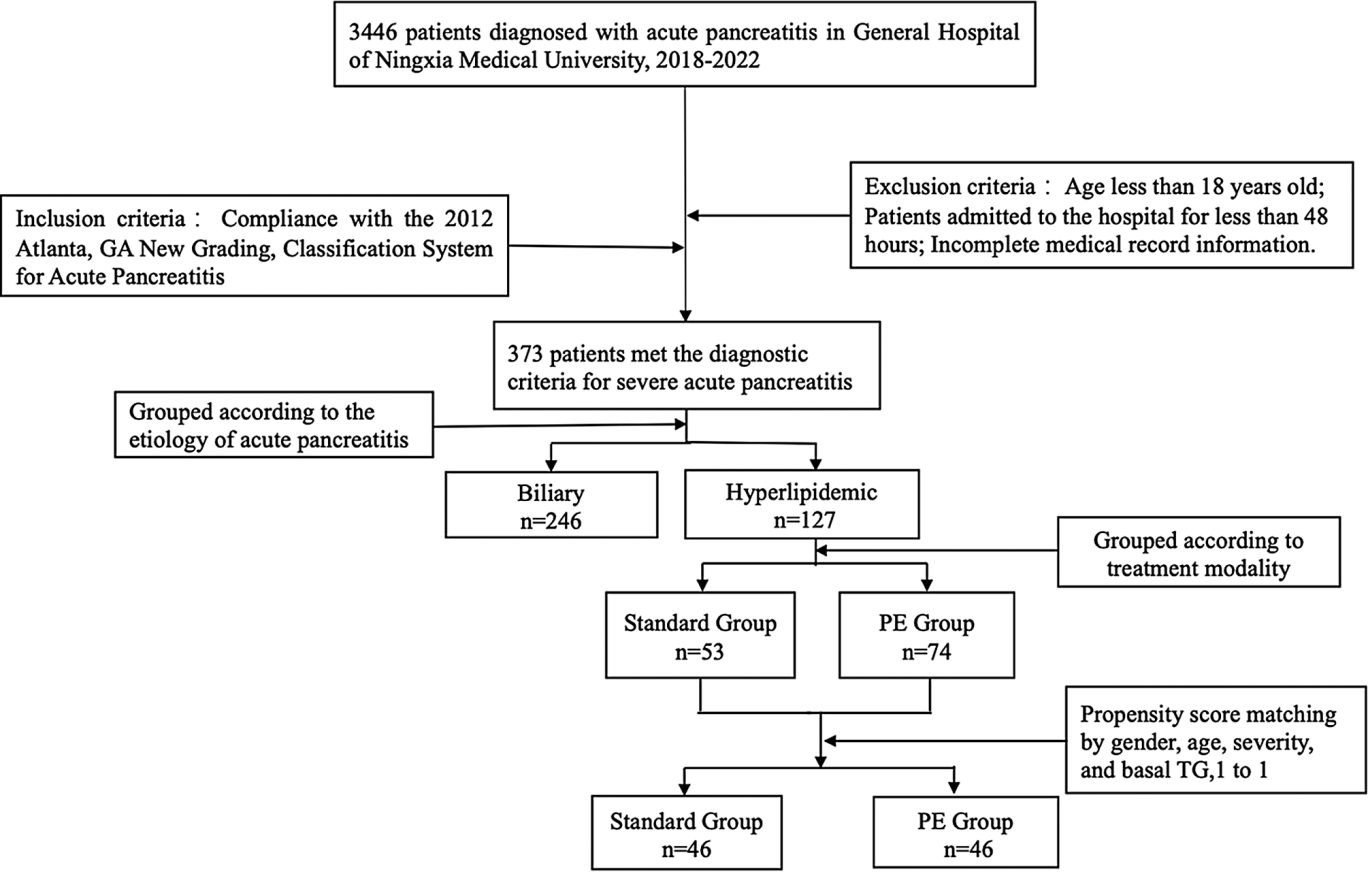
Research sample screening and grouping process

A retrospective research method was employed to include 3446 patients who were admitted to the General Hospital of Ningxia Medical University between January 2018 and December 2022 with a definitive diagnosis of acute pancreatitis. Among these patients, 373 met the inclusion criteria for inclusion in the study. After adjusting for gender, age, severity, and baseline TG using propensity score matching (PSM), 92 patients were included in the study. A comparison was made between the clinical outcomes of these 92 patients who received conventional therapy and plasma exchange therapy(Fig. 1). This study was approved by the Medical Research Ethics Review Committee of the General Hospital of Ningxia Medical University (KYLL-2023-0013).

The exclusion criteria were as follows: (1) age < 18 years, (2) admitted for less than 48 h, and (3) incomplete medical records, (4) lipid testing is not performed within 24 h of hospital admission.

### Methods

The severity grading criteria of the Revised Atlanta Types were utilized to categorize severe acute pancreatitis into biliary and hyperlipidemic groups according to etiology. Patients were divided into two groups according to treatment modality: standard treatment and plasma exchange (PE). All scores were assigned by specialists who evaluated the patient data. All patients received primary care, and the plasma exchange group was ordered to execute plasma exchange at least once within 48 h after admission. Plasma exchange plan: (1) Select plasma as the exchange solution (supplement with albumin when insufficient); (2) Each exchange of 1-1.5 times plasma volume: Use Kaplan’s formula to calculate plasma volume, with plasma volume = 0.065 ∗ body weight(kg) ∗ (1-hematocrit). The minimum exchange plasma volume is 50% of the ideal plasma exchange volume, and the portion that does not reach the perfect plasma volume is replaced with an albumin injection. PE adopts a membrane plasma separator, Plasmaflo (OP-08 W).

### Definition

The diagnostic criteria for hyperlipidemic acute pancreatitis were as follows: (1) the patient fulfilled the diagnostic criteria for acute pancreatitis according to the 2012 Atlanta, GA New Grading Classification System for Acute Pancreatitis 2a [14]; (2) the patient also had elevated levels of triglycerides, with a serum triglyceride level of at least 11.3 mmol/L; or a serum triglyceride level between 5.65 and 11.3 mmol/L in the presence of coeliac disease [15]. The diagnosis of HLAP was confirmed when both criteria (1) and (2) were satisfied.

The cause of AP is considered to be related to biliary stones when the gallbladder or bile ducts or both are found by abdominal ultrasound, CT, magnetic resonance, or endoscopic retrograde cholangiopancreatography (ERCP).

The Revised Atlanta Definitions [14] classify AP severity as mild acute pancreatitis (MAP), moderately severe acute pancreatitis (MSAP), and severe acute pancreatitis (SAP). Individuals with MAP do not experience organ failure or local or systemic consequences. MSAP is characterized by transitory organ failure (< 48 h) or local/systemic consequences without chronic organ failure. Patients with SAP experience organ failure lasting beyond 48 h.

Organ failure was defined for this study using a modified Marshall score of ≥ 2 or an APACHE II score of ≥ 8 [14].

### Data collection

Data were extracted from hospital electronic database records and patients’ medical records. Comorbidities, age, and gender were gathered. During hospitalization, the following information was documented: clinical manifestations, modified computed tomography severity index (MCTSI) score, organ failure status, laboratory and imaging data, treatments administered, intensive care unit admission, duration of stay, and prognosis. All laboratory results were obtained within 24 h of admission. Baseline triglyceride (TG) and serum amylase (AMY) levels were determined using the initial test after the attack. Serum lipid levels were assessed within 48 h of lipid-lowering treatment administration.

### Statistical analysis

GraphPad Prism 9.0 was used for graphing, and SPSS 26.0 was used for data analysis. Normally distributed measures are shown as mean ± standard deviation and analyzed using Student’s t-test or analysis of variance. Abnormal distribution data were described by median and interquartile spacing and analyzed using the Mann-Whitney U or Kruskal-Wallis tests. Absolute values and proportions represented the categorical variables using the X^2^ or Fisher exact tests. For continuous variables before and following treatment, paired t-tests were utilized. Risk factors were identified using multifactorial logistic regression analysis, each with a 95% confidence interval odds ratio. Furthermore, a one-way analysis was conducted after executing a 1–1 PSM. A difference at *P* < 0.05 was deemed to be statistically significant.

## Results

### Basic demographic features

**Fig. 2:**
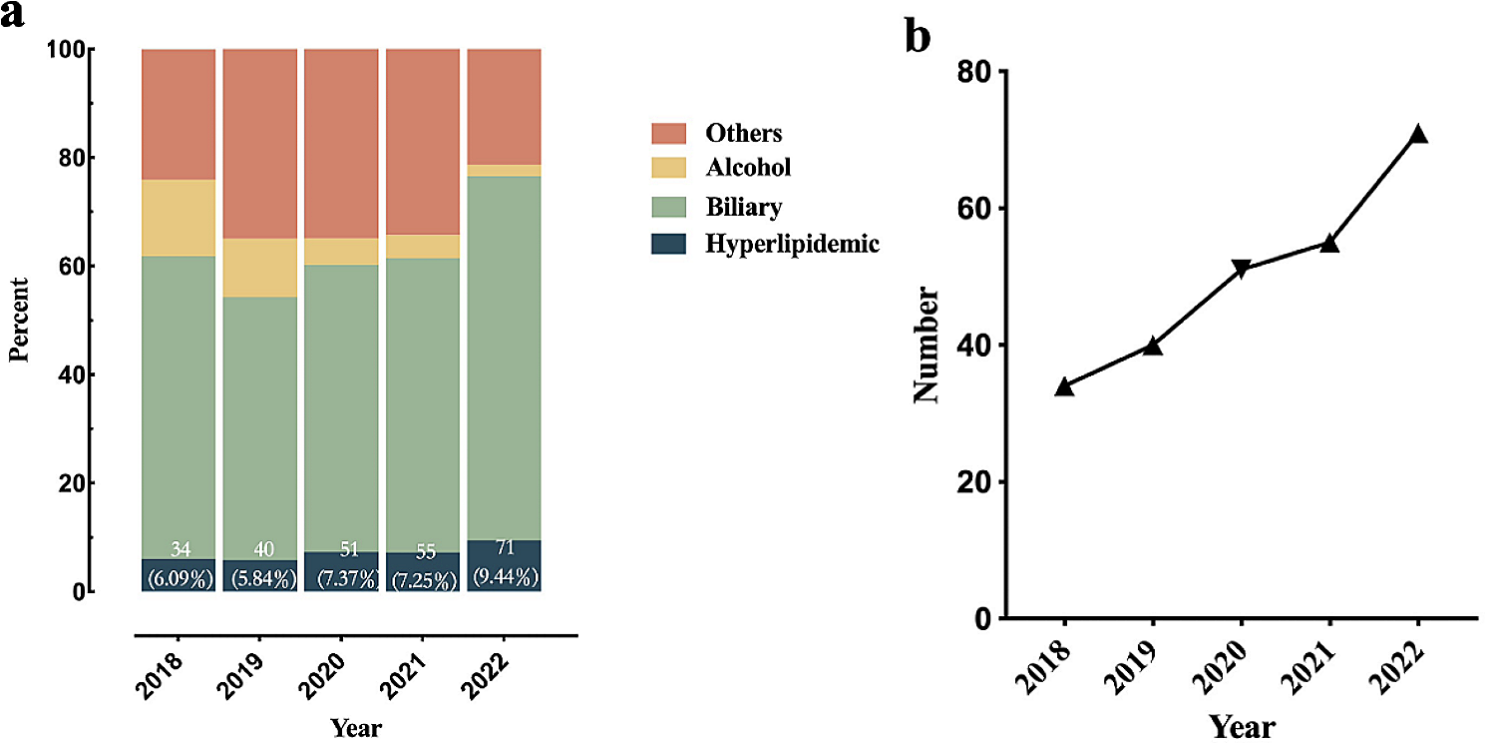
(**a**) Percentage of acute pancreatitis of different etiologies from 2018–2022. (**b**) Trends in the incidence of HLAP over 2018–2022

In the last five years, 251 patients were diagnosed with HLAP in the General Hospital of Ningxia Medical University. Research has pointed to an upward trend in the incidence of HLAP in the region. The incidence of HLAP has increased by approximately 1.6 times in the past five years, with the range of 6.09%~9.44%, while the incidence of alcoholic acute pancreatitis decreased from 14.16 to 2.13%. The study showed that hyperlipidemic has become the second leading cause of acute pancreatitis in recent years (Fig. 2).

### 5-year severe acute pancreatitis (SAP) morbidity

**Fig. 3:**
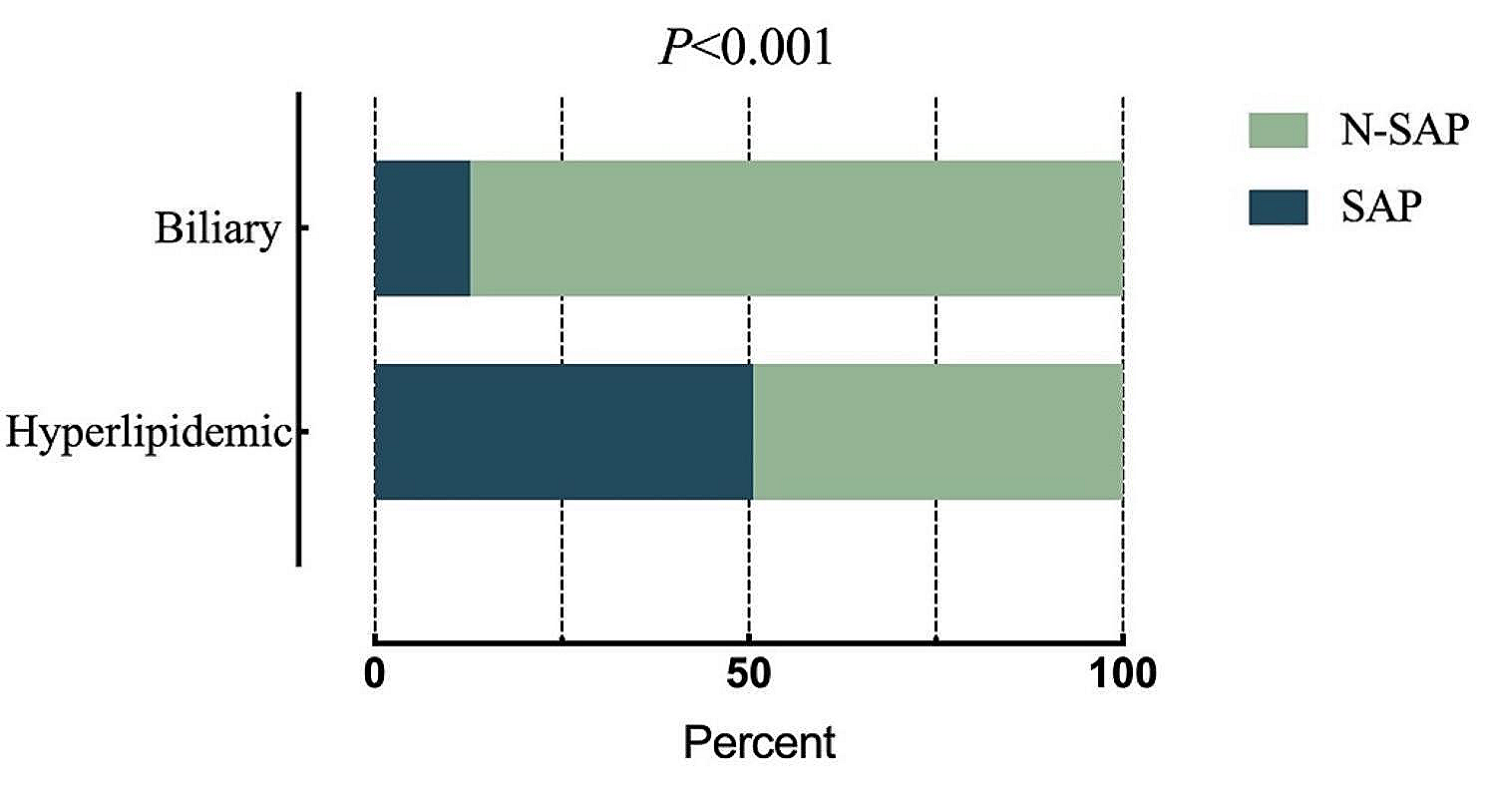
Percentage of patients with severe acute pancreatitis caused by two etiologies within five years. SAP: severe acute pancreatitis; N-SAP: Non-severe acute pancreatitis

A total of 1926 patients were diagnosed with acute biliary pancreatitis (ABP) during the period 2018–2022, of which 246 cases (12.8% of total morbidity) were SAP. A total of 251 patients were diagnosed with HLAP during the same period, of which 127 patients (50.6% of total morbidity) were diagnosed with SAP. The difference in incidence between hyperlipidemic severe acute pancreatitis (HL-SAP) and biliary severe acute pancreatitis was statistically significant over five years(*P* < 0.05) (Fig. 3).

### Clinical characteristics of HL-SAP

A total of 127 patients were diagnosed with HL-SAP. The mean age of the patients was 37.1 ± 8.7 years, and the majority were young and middle-aged (about 92.1%). The male-to-female ratio was 2.3 (89/38). The mean baseline TG was 28.25 ± 13.28 mmol/L for HL-SAP. Amylase exceeded 110 U/L in 84.3% of patients and 330 U/L in only 47.2%. Patients with HL-SAP who also had fatty liver were 83.5%. The mean Body mass index (BMI) in patients was 27.8 ± 3.3 kg/m^2^. Standardized lipid-lowering therapy without plasma exchange was used in 41.7% of patients, with plasma exchange in 58.3% and ERCP in 43.3%. The incidence of organ failure (61, 48.0%), recurrence rate (42, 33.1%), and CTSI score (5.1 ± 0.9) in patients with HL-SAP. Approximately 55.1% of patients were admitted to the ICU for intensive treatment. The average length of hospital stay for HL-SAP patients was 22.5 ± 6.6 days, the average medical cost was 64,920.73 ± 68,965.56 Chinese Yuan (CNY), and 27 (21.3%) patients died during hospitalization (Table 1).

**Table 1:** Clinical characteristics of patients with hyperlipidemic severe acute pancreatitis

Characteristic	All(*N* = 127)
Age,yr	37.1 ± 8.7
Sex, n(%)	
Male	89(70.1)
Female	38(29.9)
BMI, kg/m^2^	27.8 ± 3.3
Complications, n(%)	
Diabetes	66(52.0)
Fatty liver	106(83.5)
Hypertensive	62(48.8)
Recurrence, n(%)	42(33.1)
APACHE II	14.9 ± 6.2
CTSI	5.1 ± 0.9
Ranson	3.72 ± 1.4
Organ failure, n(%)	61(48.0)
Treatment, n(%)	
Standard treatment	53(41.7)
PE	74(58.3)
ERCP	55(43.3)
Baseline TG,mmol/L	28.25 ± 13.28
Baseline AMY, U/L > 110U/L,n(%)	539.94 ± 418.00 107(84.3)
> 330U/L,n(%)	60(47.2)
Admission to ICU,n(%)	70(55.1)
Medical costs,CNY	64920.73 ± 68965.56
Length of hospital, d	22.5 ± 6.6
Death, n(%)	27(21.3)

BMI: Body mass index; APACHE II: Acute Physiology and Chronic Health Evaluation II; CTSI: CT severity index; PE: plasma exchange; TG: Triglyceride; AMY: Amylase; ICU: Intensive care unit; ERCP: Endoscopic Retrograde Cholangio Pancreatography; CNY: Chinese Yuan

### Comparison of the general clinical information of SAP patients in the hyperlipidemic group and the biliary group

A total of 373 patients with severe acute pancreatitis met the inclusion criteria and were divided into 127 cases in the hyperlipidemic group and 246 cases in the biliary group by etiology.

Table 2 shows that there were significant differences between the two groups of patients in terms of age (*P* < 0.001), gender (*P* = 0.013), BMI (*P* < 0.001), comorbid fatty liver (*P* < 0.001), and recurrence rate (*P* < 0.001). In contrast, there was no statistically significant difference in comorbid diabetes (*P* = 0.139) between the two groups.

**Table 2:** Comparison of general clinical data between the two groups

Characteristic	Hyperlipidemic (*n* = 127)	Biliary (*n* = 246)	X^2^/Z	P
Age, yr	37.1 ± 8.7	60.7 ± 15.8	-11.936	< 0.001
Male, n(%)	89(70.1)	140(57.0)	6.128	0.013
BMI, kg/m^2^	27.82 ± 3.33	24.19 ± 1.69	-11.036	< 0.001
Complications, n(%)				
Diabetes	66(52.0)	108(43.9)	2.190	0.139
Fatty liver	106(83.5)	26(10.6)	194.651	< 0.001
Recurrence, n(%)	42(33.1)	23(9.3)	32.754	< 0.001

BMI: Body mass index

### Comparison of complications and severity during the disease in SAP patients in the hyperlipidemic group versus the biliary group

There was a statistical difference in hydrothorax, abdominal compartment syndrome (ACS), organ failure (respiratory, multiple organ dysfunction syndrome, MODS), length of hospital stay, medical costs, and death between the two groups (*P* < 0.05). In comparison, there was no statistically significant difference between the two groups for organ failure (renal, cardiovascular) and patients requiring ICU admission (*P* > 0.05). There was a statistically significant difference (*P* < 0.05) between the two groups in APACHE II score (14.9 ± 6.2 vs. 13.2 ± 4.5, *P* = 0.021. The CTSI score was significantly higher in the hyperlipidemic group than in the biliary group (5.1 ± 0.9 vs. 4.6 ± 0.7, *P* < 0.001). In contrast, there was no statistically significant difference in the Ranson score (3.72 ± 1.40 vs. 3.66 ± 1.36, *P* = 0.74) between the two groups (*P* > 0.05) (Table 3).

**Table 3:** Comparison of severity and complications between the two groups

Characteristic	Hyperlipidemic (*N* = 127)	Biliary (*N* = 246)	X^2^/Z	*P*
Hydrothorax, n(%)	47(37.0)	39(15.9)	21.128	< 0.001
ACS	27(21.3)	11(4.5)	25.801	< 0.001
Organ failure, n(%)				
Respiratory	96(75.6)	68(27.6)	78.163	< 0.001
Renal	18(14.2)	23(9.3)	1.992	0.158
Cardiovascular	26(20.5)	32(13.0)	3.554	0.059
MODS	48(37.8)	21(8.5)	47.559	< 0.001
Admission to ICU,n(%)	70(55.1)	129(52.4)	0.242	0.623
Length of hospital, d	16.1 ± 9.7	12.9 ± 10.3	-3.490	< 0.001
Medical costs,CNY	64920.73 ± 68965.56	49011.05 ± 52150.54	-2.515	0.012
APACHE II	14.9 ± 6.2	13.2 ± 4.5	-2.914	0.021
CTSI	5.1 ± 0.9	4.6 ± 0.7	-6.687	< 0.001
Ranson	3.72 ± 1.40	3.66 ± 1.36	-0.332	0.740
Death, n(%)	27(21.3)	30(12.2)	5.316	0.021

ACS: Abdominal compartment syndrome; MODS: Multiple organ dysfunction syndrome; ICU: Intensive care unit; APACHE II: Acute Physiology and Chronic Health Evaluation II; CTSI: CT severity index; CNY: Chinese Yuan

### Comparison of laboratory parameters between the two groups

Baseline TG (*P* < 0.001), baseline AMY (*P* < 0.001), creatinine (*P* = 0.009), lactate (*P* < 0.001), glucose (*P* < 0.001), D-dimer (*P* < 0.001), albumin (*P* < 0.001), LDH (*P* < 0.001), phosphorus (*P* = 0.001), calcium (*P* < 0.001), and oxygenation index (*P* = 0.024) were statistically significant (*P* < 0.05) (Table 4).

**Table 4:** Comparison of laboratory parameters between the two groups

Characteristic	Hyperlipidemic (*N* = 127)	Biliary (*N* = 246)	Z	*P*
Baseline TG,mmol/L	26(16.3,35.0)	1.6(1.1,2.1)	-15.831	< 0.001
Baseline AMY,U/L	440.8(270.8, 690.0)	712.7(238.8, 1165.5)	-3.42	< 0.001
TC,mmol/L	7.8(6.9, 11.6)	3.0(2.3, 3.9)	-15.416	< 0.001
CRP,mg/L	210.0(114.0, 282.0)	202.0(99.1, 275.0)	-1.080	0.280
Albumin,g/L	29.8(25.6, 33.8)	33.5(30.3, 37.5)	-6.210	< 0.001
TBIL, mmol/L	29.0(16.3, 117.3)	35.2(19.1, 92.8)	-1.485	0.138
LDH, U/L	659.0(510.0, 1071.0)	736.5(510.5, 1418.2)	-5.043	< 0.001
Creatinine,µmol/L	64.1 (50.3, 110.0)	59.4(45.9, 80.7)	-2.627	0.009
Lactate,mmol/L	3.0(2.1, 4.1)	1.9(1.3, 2.5)	-7.894	< 0.001
Glucose,mmol/L	13.1(9.8, 17.8)	7.6(5.6, 9.7)	-10.461	< 0.001
WBC,× 10^9^/L	10.47 (8.21,14.50)	10.20(7.10, 14.88)	-0.843	0.399
PLT,× 10^9^/L	184.0(132.0, 255.0)	187.0(141.0, 258.0)	-0.447	0.655
Hb,g/L	112.0(92.0, 130.0)	105.0(82.7, 128.0)	-1.595	0.111
HCT,%	32.3(27.8, 37.8)	34.7(29.5, 38.6)	-1.612	0.107
Phosphorus, mmol/L	0.90(0.70, 1.16)	1.03(0.83, 1.30)	-3.198	0.001
Calcium, mmol/L	1.06(0.90, 1.21)	1.86(1.14, 2.06)	-9.870	< 0.001
Oxygenation index	180.5(140.0, 224.5)	210.0(131.0, 281.0)	-2.254	0.024
D-dimer	8.03(7.43, 8.82)	5.73(4.14, 8.30)	-7.081	< 0.001

TG: Triglyceride; AMY:Amylase;TC:total cholesterol; CRP: C-reactive protein; TBIL: Total bilirubin; LDH:Lactate dehydrogenase; WBC: White blood cell; PLT: Blood platelet; Hb: Hemoglobin; HCT: Hematocrit

### Analysis of associated risk factors

Multifactorial logistic regression analysis confirmed that high BMI (*P* = 0.0038, OR = 1.336, 95%CI: 0.99–1.804), high CRP (*P* = 0.022, OR = 1.011, 95%CI: 1.003–1.019), low calcium (*P* = 0.003, OR = 0.016, 95%CI. 0.001–0.239), low albumin (*P* = 0.012, OR = 0.045, 95%CI: -0.062-0.192), and high D-dimer (*P* = 0.041, OR = 0.619, 95%CI: 0.053–2.510) were risk factors for HL-SAP (Table 5).

**Table 5:** Factors associated with HL-SAP according to multivariate logistic Regression Analysis

Variable	B	OR	95%CI	*P*
BMI	0.153	1.336	0.99–1.804	0.038
TG	0.018	1.360	1.213–1.524	0.895
AMY	0.001	1.412	0.916–2.175	0.285
CRP	0.110	1.011	1.003–1.019	0.022
Calcium	0.472	0.016	0.001–0.238	0.003
Hb	-0.005	-0.030	-0.024-0.015	0.624
Albumin	0.065	0.045	-0.062-0.192	0.012
D-dimer	1.282	0.619	0.053–2.510	0.041

OR: Odds ratio; TG: Triglyceride; AMY: Amylase; CRP: C-reactive protein; Hb: Hemoglobin

### Comparisons between standard and PE treatments

**Fig. 4:**
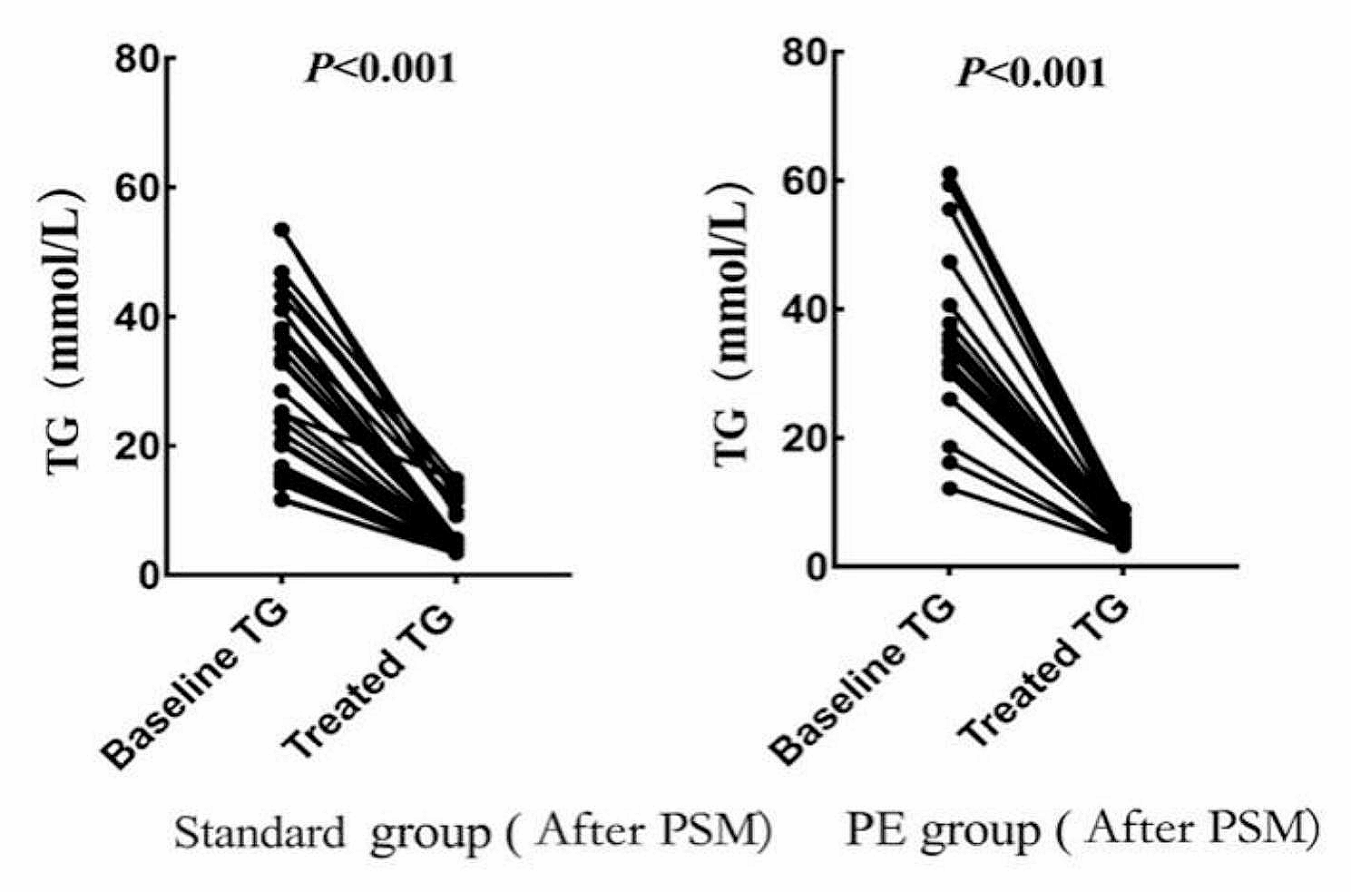
Changes in serum triglyceride levels in both groups before and after treatment. TG: Triglyceride; PE: Plasma exchange; PSM: Propensity score matching

Of the 127 patients, 53 received standard treatment, and 74 received PE. Significant differences in APACHE II (*P* = 0.001) and baseline TG (*P* = 0.004) were observed between patients treated with standard and PE treatments (Table 6). Due to disease severity, baseline TG, and other confounding factors, propensity score matching (PSM) analyses were used. PSM adjusted for gender, age, severity score, and baseline TG. After PSM, were 46 cases in the standard group and 46 cases in the PE group (1:1, matching tolerance = 0.02) (Table 6). APACHE II score (*P* = 0.786), CTSI score (*P* = 0.693), baseline TG (*P* = 0.108), length of hospital stay (*P* = 0.092), and mortality (*P* = 0.791) were not statistically significant (*P* > 0.05). In HL-SAP patients, baseline serum TG levels were significantly lower in standard (*P* < 0.001) and PE (*P* < 0.001) treatment groups within 48 h of treatment (Fig. 4). 48-hour TG clearance was 83.20%±0.0% for the standard treatment group and 84.4%±0.0% for the PE group (*P* = 0.531), indicating no significant difference between the two groups. However, the difference in medical costs (*P* = 0.039) between the two groups was statistically significant.

**Table 6:** Comparison between Standard and PE treatment

Characteristic	Entire cohort		P	PSM		*P*
	Standard group (*N* = 53)	PE group (*N* = 74)		Standard group (*N* = 46)	PE group (*N* = 46)	
Age, yr	37.1 ± 8.7	36.0 ± 8.3	0.098	38.1 ± 6.9	37.9 ± 7.1	0.63
Male, n(%)	37(69.8)	52(70.3)	0.956	25(65.7)	24(63.1)	0.811
APACHE II	14.3 ± 5.5	18.1 ± 7.6	0.001	16.5 ± 6.9	16.0 ± 6.2	0.786
CTSI	2.7 ± 1.1	3.2 ± 1.2	0.026	3.0 ± 1.2	3.1 ± 1.1	0.693
TG,mmol/L	27.1 ± 12.4	36.1 ± 14.0	0.004	26.01 ± 11.8	29.85 ± 14.11	0.108
Treated TG within 48 h, mmol/L	5.9 ± 3.5	4.6 ± 1.3	0.107	6.0 ± 3.4	5.2 ± 1.4	0.489
Clearance rate of TG within 48 h, %	76.77 ± 7.83	83.07 ± 3.93	< 0.001	83.20 ± 0.0	84.4 ± 0.0	0.531
CRP,mg/L	188.0 ± 120.4	264.3 ± 121.4	0.014	198.7 ± 145	234.2 ± 123.3	0.095
Albumin,g/L	32.6 ± 5.5	34.4 ± 4.9	0.029	34.57 ± 5.02	32.51 ± 6.59	0.088
TBIL, mmol/L	68.6 ± 66.2	58.2 ± 59.9	0.520	78.8 ± 68.2	52.4 ± 60.1	0.110
LDH, U/L	545.8 ± 349	762.9 ± 536.5	0.027	957.3 ± 703.9	743.9 ± 409.7	0.377
Creatinine,µmol/L	104.5 ± 71	77.1 ± 44.5	0.007	89.0 ± 47.7	85.4 ± 53.5	0.303
Lactate,mmol/L	5.2 ± 5	2.9 ± 1.4	< 0.001	4.8 ± 4.3	4.1 ± 1.6	0.061
Glucose,mmol/L	15.4 ± 7.2	13.3 ± 4.9	0.318	15.4 ± 7.8	13.0 ± 4.9	0.303
WBC,× 10^9^/L	10.6 ± 3.6	11.9 ± 4.5	0.138	10.9 ± 3.6	12.1 ± 4.5	0.220
PLT,× 10^9^/L	177.1 ± 83.6	214.9 ± 103	0.040	185.0 ± 79.1	213.0 ± 116.1	0.366
Hb,g/L	105.2 ± 38.4	100.5 ± 43.2	0.494	103.0 ± 41.1	108.8 ± 32.9	0.366
Phosphorus, mmol/L	0.93 ± 0.32	0.97 ± 0.40	0.604	0.96 ± 0.31	0.92 ± 0.39	0.519
Calcium, mmol/L	0.93 ± 0.32	1.1 ± 0.26	0.914	1.13 ± 0.32	1.14 ± 0.32	0.574
Oxygenation index	262 ± 151	223 ± 118	0.329	242.3 ± 146.8	215.5 ± 119.8	0.380
D-dimer	8.41 ± 0.86	7.90 ± 0.94	0.002	8.26 ± 0.86	8.19 ± 1.02	0.807
HCT,%	34.2 ± 8.0	33.5 ± 7.5	0.803	35.8 ± 8.4	34.2 ± 8.1	0.062
Length of hospital, d	20.5 ± 5.7	15.7 ± 9.6	< 0.001	19.9 ± 4.9	19.8 ± 11.1	0.092
Medical costs,CNY	38515.06 ± 34216.37	61072.88 ± 82837.10	0.025	42934.62 ± 34654.60	75377.40 ± 102191.18	0.039
Death, n(%)	16(30.2)	11(14.9)	0.037	10(26.3)	9(23.6)	0.791

PSM: Propensity score matching; APACHE II: Acute Physiology and Chronic Health Evaluation II; CTSI: CT severity index; TG: Triglyceride; CRP: C-reactive protein; TBIL: Total bilirubin; LDH: Lactate dehydrogenase; WBC: White blood cell; PLT: Blood platelet; Hb: Hemoglobin; HCT: Hematocrit; CNY: Chinese Yuan

## Discussion

Acute pancreatitis is a pancreatic inflammatory disease with a mortality rate of up to 20–25% in severe instances [16]. AP is the leading cause of hospitalization and emergency room visits in the United States and many other nations, with an estimated yearly hospitalization cost of $2.6 billion in the United States [17]. The cause of acute pancreatitis varies by location. Extensive retrospective investigations from Ireland and the United States revealed a 1:1 ratio of alcoholic to gallstone pancreatitis, with each etiology accounting for 23–36% of AP cases in these countries [18]. AP due to gallstones were discovered to be substantially more common in southern European countries than AP due to alcohol, according to a European study [19]. However, HLAP has been rapidly developing throughout Asia in recent years. In Denmark, the standardized incidence of HLAP episodes increases from 0.7 per 100,000 population years to 1.7 per 100,000 from 2008 to 2019, representing a 2.4-fold increase in incidence during the previous ten years [20]. In Taiwan, the frequency of hyperlipidemic as a cause of AP was reported to be 6.3–12.3% [21]. Consistent with the previous studies, the prevalence of HLAP in this research has increased around 1.6-fold in the last five years, ranging from 6.09 to 9.44%. Hyperlipidemic has now surpassed alcohol as the second cause of pancreatitis development. This phenomenon may be related to the rapid economic growth in Northwest China in recent years and changes in people’s dietary structure and lifestyles. High-fat diets, overweight, and poor glycaemic control affect triglyceride levels, leading to fatty liver and HALP. Furthermore, improved medical care has increased the diagnostic efficiency of this disease.

The prognosis and severity of disease in patients with AP associated with hyperlipidemic are subjects of debate. Some reports imply that the progression of AP caused by hyperlipidemic is more severe than other causes [22]. In contrast, others indicate that the disparity in disease severity is not statistically significant [23]. Nevertheless, the lack of consistency among these studies (regarding case selection, diagnostic criteria, sample size, and triglyceride cutoff values) obscures the true nature of pancreatitis associated with hyperlipidemic. The data from this study show that hyperlipidemic acute pancreatitis was associated with a higher risk of MODS (37.8%) and respiratory failure (75.6%), as well as more extended hospitalization, higher healthcare costs, and a poorer prognosis compared to biliary pancreatitis. This study found that patients with HLAP were more likely to develop severe acute pancreatitis (50.60% vs. 12.77%, *P* < 0.001). Therefore, early prediction of severe pancreatitis is essential. CRP, calcium, and albumin are predictive factors for severe acute pancreatitis. Yu et al. [24]study confirmed that low calcium is associated with developing HL-SAP. A retrospective study demonstrated that high CRP and low albumin are risk factors for moderate to HL-SAP [25]. Whereas high BMI and high D-dimer were unique risk factors for HL-SAP in the present study, Guo et al. [26]showed that D-dimer is a sensitive predictor of HLAP severity. Therefore, early diagnosis and intervention treatment are essential.

Safe and effective lipid-lowering therapy is essential for patients with HL-SAP. Fasting, lipid-lowering medications, including fibrates, insulin, and heparin, as well as blood purification techniques like hemofiltration, hemoperfusion, double-filtration plasmapheresis, PE, and other blood therapies, are often used in lipid-lowering treatments. There is still controversy about whether patients admitted with HL-SAP can benefit from plasma exchange. In an observational study, Christian JB et al. found that conventional therapies were also effective in decreasing lipids and had the advantage of being non-invasive and low-cost [27]. Conversely, blood purification has the disadvantages of being invasive and expensive. The current study compared PE and standard therapy’s effects on lowering lipids and patient prognosis. The results showed that TG levels were significantly reduced in the standard treatment group (*P* < 0.001) and the PE group (*P* < 0.001) at 48 h. Compared to conventional therapy, PE has no advantage in clearing serum TG (83.20% ± 0.0% vs. 84.4% ± 0.0%, *P* = 0.531). There were no significant differences between the two groups regarding the length of hospitalization (19.9 ± 4.9 vs. 19.8 ± 11.1, *P* = 0.092) and mortality (26.3% vs. 23.6, *P* = 0.791). However, compared to plasma exchange, the standard treatment is low-cost. In summary, this investigation demonstrated that PE did not shorten the course of the disease, reduce the incidence of organ failure, or improve the prognosis of the patients. In a small retrospective study conducted in Japan, Miyamoto K. et al. noted that PE did not reduce TG levels faster or improve prognosis compared to patients who did not undergo PE and, therefore, did not support the use of PE [28].

In conclusion, this retrospective study confirms that hyperlipidemic is the second leading cause of acute pancreatitis and is markedly severe by summarising clinical profile, severity, and associated risk factors. Furthermore, plasma exchange does not improve clinical outcomes in HL-SAP and is costly, whereas standardized treatments are equally safe, effective, and low-cost in early treatment.

### Study strengths and limitations

The study’s strengths include the precision of the data due to rigorous inclusion and exclusion criteria and the use of PSM to eliminate testing errors. There are few studies detailing how to choose a treatment modality for HL-SAP, which speaks to the innovation of this study.

There were also limitations to this study. The present study used a single-centre retrospective design. The prevalence of HLAP varies geographically, so this research only reflects the prevalence in a particular region. Multicentre, more extensive sample size statistical evaluations still need to be included.

## Conclusion

The incidence of HLAP exhibited a significant increase, remarkable severity, recurrent trend, and mortality. High BMI, high CRP, low calcium, low albumin, and high D-dimer are risk factors for HL-SAP. Plasma exchange does not improve the prognosis of patients with HL-SAP compared with standardized therapy. Therefore, standardized therapy in early clinical management is equally effective, safe, and low-cost in early treatment.

## Data Availability

All data are contained in the article. The raw data will be shared upon request. Contact the corresponding author.

## Abbreviations

HLAP: Hyperlipaemic acute pancreatitis
TG: Triglyceride
HL-SAP: Hyperlipaemic severe acute pancreatitis
ACS: Abdominal compartment syndrome
MODS: Multiple organ dysfunction syndrome
CRP: C-reactive protein
OR: Odds ratio
BMI: Body mass index
PSM: Propensity score matching
AP: Acute pancreatitis
FFA: Free fatty acid
PE: Plasma exchange
APACHE II: Acute Physiology and Chronic Health Evaluation
MCTSI: Modified Computed Tomography Severity Index
MAP: Mild acute pancreatitis
MSAP: Moderately severe acute pancreatitis
SAP: Severe acute pancreatitis
AMY: Amylase
ABP: Acute biliary pancreatitis
ERCP: Endoscopic Retrograde Cholangio Pancreatography
CRRT: Continuous renal replacement therapy
TC: Total cholesterol
TBIL: Total bilirubin
LDH: Lactate dehydrogenase
WBC: White blood cell
PLT: Blood platelet
Hb: Hemoglobin
HCT: Hematocrit
